# Enzyme-Embedded Biodegradable Plastic for Sustainable Applications: Advances, Challenges, and Perspectives

**DOI:** 10.1021/acsabm.4c01628

**Published:** 2025-02-13

**Authors:** Shengwei Sun

**Affiliations:** School of Engineering Sciences in Chemistry, Biotechnology and Health, Department of Fibre and Polymer Technology, KTH Royal Institute of Technology, 10044 Stockholm, Sweden; School of Engineering Sciences in Chemistry, Biotechnology and Health, Science for Life Laboratory, Tomtebodavägen 23, 17165 Solna, Sweden

**Keywords:** enzyme, biodegradable plastics, enzyme-embedded BPs, biodegradation, challenges

## Abstract

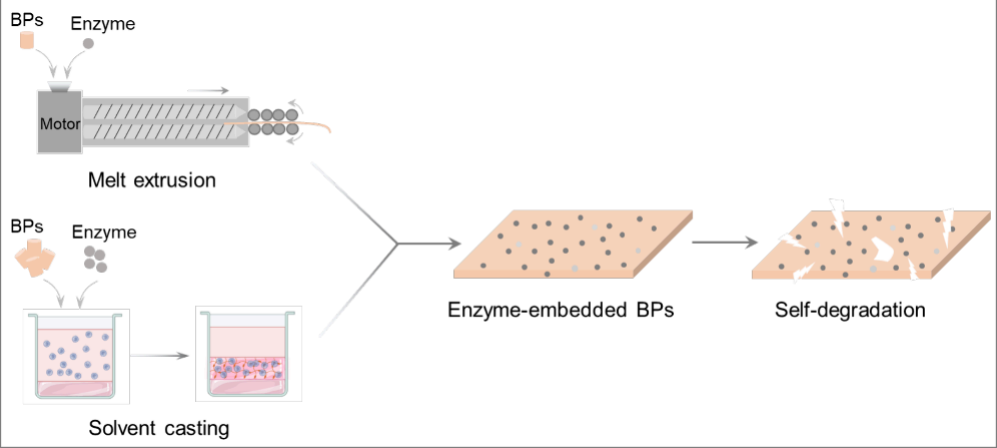

Global plastic production is increasing yearly, with packaging materials and disposable plastics accounting for a sizable portion of the total. Despite its apparent advantages, the resulting plastic waste accumulates in landfills and oceans, causing severe environmental and public health issues. Shifting from conventional plastics to biodegradable plastics (BPs) is increasingly being proposed as an efficient management of end-of-life plastics. While several BPs such as poly(lactic acid), poly(ε-caprolactone), and poly(hydroxyalkanoates) have been widely used, their biodegradation rates often do not meet the anticipated level under home-compost or other certain environments (e.g., soil, marine). Recently, enzyme-embedded BPs have emerged as an outstanding alternative to currently used synthetic plastics. It achieves rapid degradation and compostability by introducing a specific enzyme into the biodegradable polymer. In this context, this review aims to summarize the recent advances in the development of such superior biomaterials. It identifies and prioritizes the critical success factors required for the production of enzyme-embedded BPs. The review also discusses several challenges in the development and application of these innovative polymer materials.

## Introduction

Plastics consist of very large molecules (called polymers) and are a broad class of synthetic or semisynthetic materials. They came to prominence in the early 20th century. Currently, plastics are ubiquitous in our daily lives. Their excellent characteristics (e.g., adaptability, being lightweight, durable, nontoxic, flexible, and inexpensive to produce) drive the rapid growth of global plastic production and consumption. The cumulative plastic production is forecast to reach trillions of metric tons by 2050.^1^ Nevertheless, the metal-like durability once considered an advantage of plastic has resulted in a massive accumulation of plastic wastes in the terrestrial and aquatic ecosystems.^2^ This is one of the most pressing environmental issues worldwide, seriously threatening human and animal health. While some measures have been or are being implemented to reduce the plastic footprint, recycling and upcycling remain the most effective ways to deal with these white wastes. Unfortunately, only about 9% of postconsumer plastics are eventually recycled.^3^

Biodegradable plastics (BPs) that can be decomposed by specific depolymerases secreted from environmental microorganisms may serve as a promising alternative to conventional plastics.^4^ Different types of BPs are being continually developed and produced in industries, such as poly(lactic acid) (PLA), poly(ε-caprolactone) (PCL), and poly(hydroxyalkanoates) (PHAs). They are widely used in applications including single-use plastic bags, disposable tableware, and agricultural plastic mulch films.^5^ However, challenges remain in achieving sustainable plastic waste management. The BP products still suffer from low degradation efficiency in open environments and under anaerobic conditions.^6^ For example, PLA is generally recognized as a biodegradable polymer, but it can only be effectively degraded under industrial composting conditions at 60 °C or higher. It exhibits very low biodegradability in nature (e.g., soil and aquatic environments) and under mild conditions such as home composting.^7−9^ Additionally, the micro- and nanoplastics generated from BPs are emerging as contaminants that have shown detrimental effects across the various ecosystems.^10^ Hypothetically, if BP polymer degradation is intentionally promoted following its application, the resulting oligomer can be taken up by microorganisms in the environment, thus facilitating BP-derived carbon cycling and preventing massive accumulation, a highly desirable outcome.

For this purpose, enzyme-embedded BPs represent a new type of biomaterial with the ability to self-degrade by loading known and validated enzymes into biodegradable polymers.^11^ Common types of polymer hydrolases include lipases, cutinases, esterases, amylases, and proteases, which can be introduced through solvent-casting or melt-extrusion procedures.^12^ It utilizes enzyme catalysis as the primary mechanism to accelerate the degradation process of polymers from the inside to the outside. Developing such “smart” polymer materials holds the promise of rapid, on-demand automatic degradation of plastics in nature after use. Recently, many efforts have been made to develop these novel biomaterials. By incorporating enzyme engineering, polymer chemistry and process engineering, enzyme-embedded PLA and PCL have been regarded as one of the most successful examples.^11,13^ However, the technical optimization and the scaling-up application of these innovative biomaterials are still a long way off.

**Figure 1:**
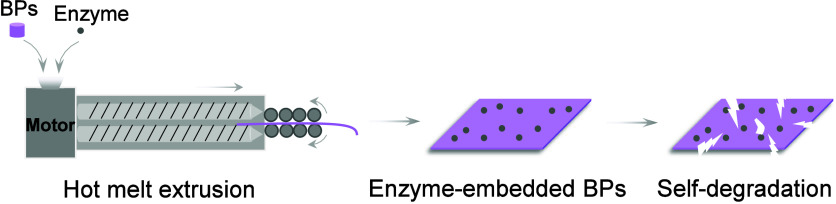
Representative diagrams of the production of enzyme-embedded BPs. In short, the BPs and enzyme are premixed and then hot-melt-extruded. After cooling, the resulting composite materials are self-degradable.

This review aims to highlight the great potential of enzyme-embedded BPs for sustainable applications (Figure 1). It summarizes a wide range of reported enzymes that could be applied in collaboration with BPs. Then it describes current advances in the research and development of enzyme-embedded BPs. The review also discusses the key factors required for the processing and production of this composite. It further points out several challenges in the development and application process. The implications and future directions for these emerging biomaterials are also described.

## Enzyme Discovery for BP Degradation

**Figure 2:**
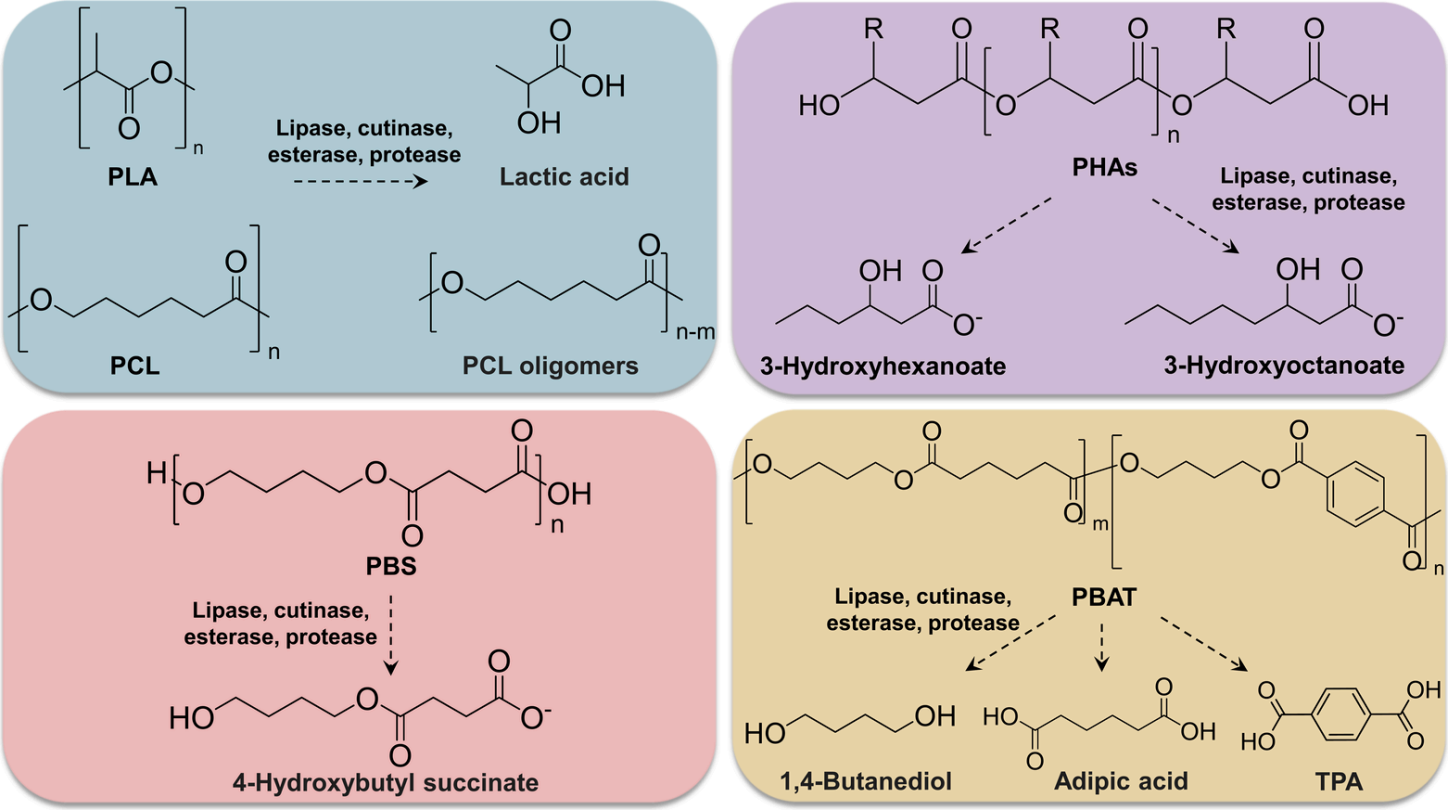
Schematic diagrams of the effects of various enzymes on the BPs. Polymers including PLA, PCL, PHAs, PBS, and PBAT can be enzymatically degraded into their monomers or oligomers. Some representative degradation products are described.

Identification and characterization of potential enzymes for effective polymer degradation are key prerequisites before developing BPs with embedded enzymes. At present, commonly used biodegradable polymers include PLA, PCL, PHAs, poly(butylene succinate) (PBS), and poly(butylene terephthalate/adipate) (PBAT). Microbial enzyme-mediated degradation of BPs has been long reported. Various types of hydrolases including esterases, lipases, cutinases, and proteases have been shown to have great potential for degrading BPs (Figure 2). Both esterases (EC 3.1.1.1, carboxyl ester hydrolases) and lipases (EC 3.1.1.3, triacylglycerol hydrolases) are generally defined as the enzymes that can catalyze the hydrolysis of ester bonds in the polyester-based BPs, which have been differentiated on the basis of their substrate specificity. Cutinases (EC 3.1.1.74) are a subclass of esterase enzymes that were initially discovered to hydrolyze the ester bonds of the plant polymer cutin, which have gained increasing importance due to their capacity to hydrolyze polyesters with a high molar mass. While proteases (EC 3.4) are able to hydrolyze amide bonds in peptide and protein substrates, they can also catalyze the hydrolysis of various polymers containing ester bonds, such as PLA, making them attractive biocatalysts for plastics recycling. A majority of these have been identified and purified from environmental microbial cultures, and others have been expressed recombinantly in *Escherichia coli* or other bacterial and yeast expression systems. Table 1 presents a summary of the reported BP-degrading enzymes, including the types of BPs and enzymes, microbial origin, reaction conditions, and degradation products.

**Table 1:** Representative BP-Degrading Enzymes Reported

BPs	enzymes	microbial origin	reaction conditions	products	ref
PLA	proteinase K	*Tritirachium album*	37 °C, pH 8.0	l-lactic acid monomers and oligomers	(14)
	PLAse	*Amycolatopsisorientalis* ssp. *orientalis*	50 °C, pH 7.0	l-lactic acid monomers	(15)
	PLD	*Amycolatopsis* sp. strain K104–1	37 °C, pH 7.0	lactic acid monomers and PLA residues	(16)
	protease LP175	*Laceyella sacchari* LP175	60 °C, pH 9.0	PLA residues	(17)
	BpAprE	*Bacillus pumilus*	30 °C, pH 8.0	l-lactic acid monomers	(18)
	cutinase-like CLE	*Cryptococcus* sp. S-2	30 °C, pH 8.0	PLA residues	(19)
	PAE	*Pseudozyma antarctica* JCM 10317	30 °C, pH 8.8	d- and l-lactic acid monomers and PLA residues	(20)
	CfCLE	*Cryptococcus flavus* GB-1	30 °C, pH 7.8	lactic acid	(21)
	Thc_Cut1 and Thc_Cut2	*Thermobifida cellulosilytica*	37 °C, pH 7.0	lactic acid	(22)
	depolymerase PlaA	*Paenibacillus amylolyticus* TB-13	37 °C, pH 10.0	lactic acid monomers and oligomers	(23)
	lipase DS04-T	*Pseudomonas* sp. DS04-T	50 °C, pH 8.0	lactic acid monomers	(24)
	lipase MTCC 2594	*Aspergillus niger* MTCC 2594	30 °C, pH 7.0	PLA residues	(25)
	esterase PlaM	Metagenome	30 °C, pH 7.0	PLA residues	(26)
	carboxylesterase ABO2449	*Alcanivorax borkumensis*	30 °C, pH 8.0	PLA residues	(27)
	esterase TKU015	*Pseudomonas tamsuii* TKU015	50 °C, pH 7.0	lactic acid monomers	(28)
	esterase GEN0105	Metagenome	30 °C, pH 8.0	lactic acid monomers and oligomers	(29)
	esterase	*Pseudomonas aeruginosa* S3	37 °C, pH 8.0	lactic acid oligomers	(30)
PCL	cutinase	*Pseudozyma japonica*-Y7–09	30 °C, pH 8.0	not mentioned	(31)
	cutinase	*Fusarium solani*	37 °C, pH 7.2	not mentioned	(32)
	lipase	*Pseudomonas* sp.	37 °C	not mentioned	(33)
	lipase	*Candida rugosa, Mucor miehei* and *Rhizopus delemar*	37 °C, pH 7.7	not mentioned	(34)
	lipase	*Fervidobacterium nodosum*	60 °C, pH 8.0	PCL monomers	(35)
	lipase	*Lactobacillus* sps.	37 °C, pH 8.1	not mentioned	(36)
	lipase CA	*Candida antarctica*	40 °C	cyclic dicaprolactone	(37), (38)
	PCLase I and PCLase II	*Pseudomonas hydrolytica* sp. DSWY01^T^	50 and 40 °C, pH 9.0 and 10.0	PCL monomers and oligomers	(39)
	esterase	*Archaeoglobus fulgidus*	80 °C, pH 8.0	PCL monomers	(40)
	PCL-degrading enzyme	*Streptomyces thermoviolaceus* subsp. *thermoviolaceus isolate* 76T-2	45 °C, pH 7.2	not mentioned	(41)
PHAs	lipase	*Bacillus subtilis* DI2	40 °C, pH 8.0	not mentioned	(42)
	lipase	*Pseudomonas chlororaphis* and *Acinetobacter lwoffii*	30 °C	3-hydroxyhexanoate, 3-hydroxyoctanoate, and 3-hydroxydecanoate monomers	(43)
	lipase	*Priestia megatarium* POD1	55 °C, pH 8.0	not mentioned	(44)
	depolymerase	*Streptomyces exfoliatus*	21 ± 1 °C, pH 8.0/10.0	not mentioned	(45)
PBS	cutinase	*Fusarium solani*	50 °C, pH 8.0	not mentioned	(46)
	lipase	*Candida antarctica*	45 °C, pH 7.2	not mentioned	(47)
PBAT	esterase PpEst	*Pseudomonas pseudoalcaligenes*	50, 65, or 80 °C, pH 7.0	not mentioned	(48)
	lipase	*Stenotrophomonas* sp. YCJ1	37 °C, pH 7.5	terephthalic acid (TPA), 1,4-butanediol, and adipic acid	(49)
	lipase	*Candida antarctica*	45 °C, pH 7.2	not mentioned	(50)
	cutinase	*Thermobifida fusca*	70 °C	TPA	(51)
	P450 (CYP) monooxygenase	*Purpureocillium lilacinum*	30 °C	not mentioned	(52)
	depolymerase	*Roseibium aggregatum* ZY-1	30 °C	oligomers (TB and BTB) and monomers (T and A)	(53)

During the past few decades, nature has evolved a variety of enzymes for the effective elimination and sustainable management of plastic waste. As demonstrated above, lipase and cutinase play a central role in the biodegradation of different types of BPs. Lipases that have been identified from bacteria and fungi are of particular interest as they show high degradation activity and broad substrate selectivity.^54^ They can catalyze a variety of reactions, including ester hydrolysis, esterification, transesterification, ammonolysis, and alcoholysis making lipases quite important in industrial applications. The monomeric building blocks produced during the enzymatic BP depolymerization process can be further used as valuable raw feedstocks realizing a circular economy.^55^ Although biodegradation of BPs offers significant advantages over chemical degradation or physical recycling, there are still several challenges that need to be addressed. First, since biodegradation of BPs is an emerging field, the number of active enzymes is limited, and those that have been reported tend to have low activity and stability. In most cases, the complete degradation of BPs has not yet been achieved. Second, BPs can be decomposed or degraded by microorganisms in the environment, but the degradation efficiency is sometimes low. Industrial composting allows for efficient enzymatic recycling and upcycling of BPs but is difficult to apply to other real-world environments such as soils and marines. Moreover, the home composting is less efficient in terms of degradation. These suggest that BP biodegradation is quite dependent on the environmental conditions (e.g., temperature, pH). Third, microplastics are usually produced when BPs enter the environment and undergo natural decomposition, which can be ingested by marine and aquatic organisms. Microplastics are now recognized as one of the most important ongoing issues.^56,57^ They pose a huge threat to human health, producing cytotoxicity, hypersensitivity, acute responses, and unwanted immune responses. These do not meet the needs of global sustainable development. Therefore, there is an immense need to develop alternative advanced materials with desirable properties, such as self-degradation.

## Current Advances in Enzyme-Embedded BPs

Recently, attempts have been made to create enzyme-embedded BPs given the discovery of a variety of BP-degrading enzymes and the quest to accelerate BP degradation. In general, innovative enzyme-embedded BP materials are made from a mixture of enzymes and polymers through a solvent-casting or hot-extrusion process. It provides faster degradation rates in buffers, tap water, and various composting conditions and reduces the release of microplastics without compromising material properties (e.g., mechanical strength). Given the limited information on enzyme-embedded BPs in the literature, this review aims to provide current knowledge about the fabrication, characterization, and application of several reported enzyme-embedded BPs (Table 2).

**Table 2:** Representative Advances in the Development of Enzyme-Embedded BPs

polymer	enzyme	preparation method	result	ref
PCL	lipase	solvent-casting method (enzyme and PCL in chloroform)	PCL films incubated for 8 days were found to be crumbled, indicating efficient polymer degradation through this enzyme-embedded approach.	(58)
PLA	proteinase K	solvent casting of PLA film and PLA extruded films at 200 °C	The rate of embedded-enzyme degradation was accelerated by dividing the film into smaller pieces.	(59)
PBS, PBSA, and PCL	lipase	melt extrusion at 130 °C (PBS), 100 °C (PBSA), or 90 °C (PCL)	Even low concentrations of these lipases embedded in the polyester films resulted in significant degradation after a short time.	(60)
PBSA	cutinase	PBSA with 5 wt % immobilized cutinase (LDH/Cut-3.4/1) prepared by compression molding (120 °C, 1 min, 10000 Psi).	The cutinase-embedded PBSA film completely degraded within 24 h.	(61)
PCL and PLA	lipase and proteinase K	solution-casting films (PCL or PLA was dissolved in toluene or dichloromethane)	PCL and PLA containing less than 2% enzymes were depolymerized in days, with up to 98% polymer-to-small-molecule conversion in standard soil composts and household tap water.	(13)
PCL	lipase	3D printing at 90 °C	The degradation rates of PCL/lipase composites were faster than lipase applied externally in the buffer.	(62)
PCL	lipase	3D printing of engineered spores and PCL matrix at 120 °C	The BC-lipase released by the germinated spore cells caused near-complete depolymerization of the polymer matrix.	(63)
PLA, PBAT, and PBS	*Candida antarctica* lipase B (CalB)	melt-extrusion/hot-pressing procedure at 130 °C (PBS), 100 °C (PBSA), and 90 °C (PCL)	CalB showed a preference for PBS films over PBAT and PLA. The self-degradable films obtained from the blends showed slow degradation.	(64)
PLA	a new PLA depolymerase (PLAase)	liquid enzyme incorporated into PCL through melt extrusion at 70 °C, forming a masterbatch, which was integrated into PLA by melt extrusion at 160 °C	The enzyme-embedded PLA film (0.02% w/w enzyme) fully disintegrated under home-compost conditions within 20–24 weeks.	(11)

For example, Khan et al. investigated the degradation of enzyme-embedded polymers based on a lipase obtained from *Lactobacillus plantarum* and PCL as a semicrystalline polymer.^58^ The enzyme-embedded PCL films were fabricated using solvent casting by adding lyophilized lipase powders (2, 4, 6, and 8 wt %) to PCL solution (1% w/v PCL chloroform solution) in Petri dishes and drying overnight. Thermogravimetric analysis revealed a significant decrease in the polymers’ thermostability with increasing lipase concentration and incubation days. Moreover, the differential thermal analysis suggested that the percent crystallinity of the remaining PCL films increased with the progression of enzyme degradation because of the effective action of lipase on the films’ amorphous regions. The weight loss percentage of PCL films increased with the increase in enzyme loading and incubation days. The 2% lipase-embedded polymer films yielded an 11% mass loss after 2 days of incubation, whereas the 8% lipase loading caused up to 73% mass loss after 8 days of incubation. Scanning electron microscopy (SEM) showed that micron-sized pores were visible after samples were incubated in buffer solution (pH 8.1) for 4 and 6 days, and PCL films incubated for 8 days appeared to be crumbled, indicating that the polymer could be effectively degraded by this enzyme embedding method. The efficient enzymatic degradation of the polymers was further confirmed by gel permeation chromatography analysis, where the number-average molecular weight of the 8% lipase-embedded PCL film was drastically reduced from 36500 to 17260.

**Figure 3:**
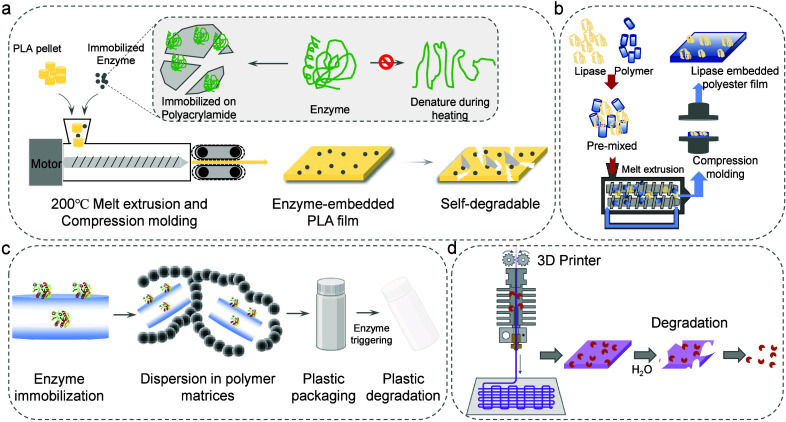
Development and production of enzyme-embedded BPs. (a) Schematic diagram of enzymatic self-biodegradation of PLA films by embedded immobilized proteinase K. Reprinted and adapted with permission from ref (59). Copyright 2020 American Chemical Society. (b) Development of self-degradable polyesters by embedding lipases through the melt-extrusion process. Reprinted and adapted with permission from ref (60). Copyright 2021 Elsevier. (c) Schematic representation of embedded-immobilized enzyme into PBSA polymer. Reprinted and adapted with permission from ref (61). Copyright 2023 Elsevier. (d) Scheme of 3D-printed enzyme-embedded polyester plastics. Reprinted and adapted with permission from ref (62). Copyright 2021 American Chemical Society.

Huang et al. demonstrated a new proteinase-embedded PLA material that can be self-degraded.^59^ Solution-cast PLA films embedded with protease K displayed a weight loss of 78% after 96 h of incubation in Tris-HCl buffer at 37 °C with shaking. SEM showed that water molecules penetrated the polymer surface and contacted the embedded enzymes, allowing continuous enzymatic degradation. Moreover, the extruded PLA films containing immobilized protease K in polyacrylamide were also prepared at 200 °C (Figure 3a). The immobilized protease K maintained its degradation activity against PLA film from the inside to form small holes and cavities. Compared to PLA extruded films with 0.5 wt % of nonimmobilized proteinase K, composite films in buffer solution showed a dramatic 2.5-fold increase in weight loss after 504 h of incubation. Moreover, the weight loss of PLA extruded films with externally added proteinase K reached saturation after 24 h of degradation time, while the weight loss of PLA extruded films with embedded proteinase K gradually increased until 504 h. These suggested that immobilization was a useful procedure for producing enzyme-embedded thermoforms. Subsequently, in another study, they selected four distinctive lipases (Lipozyme CalB L, Lipase G Amano 50, Lipase AK Amano, and Lipase PS Amano SD) and embedded them into polymer films using a hot-melt-extrusion method^60^ (Figure 3b). Given that these lipases have shown strong thermal stability and exhibited remarkable degradation activity against aliphatic biodegradable polyesters (PCL, PBSA, and PBS), even low concentrations of these lipases led to significant film degradation after a short period of time. Polymer films embedded with CalB powder showed 100% weight loss after 6 h, while enzyme-free extruded films showed little change. The films displayed significant shrinkage and rapidly disintegrated into small pieces after 3 h of degradation, which could not be recovered after 6 h. PCL films with lipase AK and lipase PS showed 17% and 60% weight loss after 96 h, while lipase G and lipase-free films remained almost unchanged. The CalB lipase-embedded PBSA extruded film lost 100% weight in the 96-h degradation experiment, and the nonlipase-embedded control sample did not degrade at all. The PBSA films embedded with lipase PS, lipase G, and lipase AK all had weight losses below 10 wt %. There was a significant difference between the weight loss obtained for each lipase and the weight loss of pure PBSA films with externally added lipase. After the 504-h degradation test, the weight loss of the PBS extruded film embedded with lipase was about 20 wt %, while the film without lipase was not degraded at all. The mechanical properties of lipase-containing films remained essentially unchanged, except for a decrease in elongation at break. The elongation at break of pure PBS was 405%, while that of the specimen containing 0.07% CalB powder decreased to 137%. Overall, these lipase-embedded materials maintained excellent physical properties, with tensile strength and Young’s modulus almost unchanged.

Romano et al. reported a polyester-degrading cutinase from *Humicola insolens*, which was immobilized by a Mg/Al layered double hydroxide structure for thermal protection^61^ (Figure 3c). Compared to the free enzyme, the thermal stability of immobilized cutinase was significantly improved, with 6 times longer than its half-life at 90 °C, and high retention of activity (>60%) after a short exposure at 200 °C. Moreover, the results showed that PBSA films with immobilized cutinase (5 wt %), were completely degraded within 24 h in sodium phosphate buffer. Approximately 0.12 mg/mL and 75.5 U/mL of cutinase were found in the degradation solution. These values correspond to approximately 34% and 16% release of protein amount and enzyme activity, respectively, from the PBSA films. In addition, there was no decrease in the weight of the films after they were stored under vacuum at room temperature for 24 h, which highlights the fact that polyester does not degrade before the enzyme is triggered. Likewise, DelRe et al. showed that semicrystalline polyesters including PCL and PLA could be self-degraded by introducing nanoscopically dispersing enzymes (*Candida antarctica* lipase B (CA-lipase), Amano PS lipase from *Burkholderia cepacia* (BC-lipase), and proteinase K from *Tritirachium album*).^13^ PCL and PLA containing less than 2% (w/w) enzyme were depolymerized within a few days. When embedded with 1.5 wt % proteinase K and 3 wt % random heteropolymer (RHP), approximately 80 wt % of the PLA depolymerized within 1 week at 37 °C in a buffer. The conversions of polymer-to-small molecule reached as high as 98% in standard soil composts and household tap water. Both enzyme-embedded PCL and PLA exhibited accelerated biodegradation in industrial soil composting (2 days for PCL at 40 °C and 6 days for PLA at 50 °C). Even in tap water or a custom-made compost setup, the PCL-RHP-lipase exhibited up to 40% and 70% mass loss after 2 and 4 days, respectively. For PLA-RHP-Protease K, mass losses of about 34% for 40 kDa PLA and 8% for 85–160 kDa PLA were observed after 5 days in soil compost at 50 °C. Moreover, the mechanical properties of PCL changed by less than 10% at 2 wt % lipase content. The tensile strength and elastic modulus of lipase-embedded PCL were like those of low-density polyethylene (LDPE).

In order to simulate or fabricate a pilot-scale thermoplastic process on the lab bench, Greene et al. reported the production and degradation of PCL/Amano lipase (AL) composites using thermal 3D printing techniques^62^ (Figure 3d). Impressively, the solid-state AL was able to withstand *in situ* processing temperatures up to 130 °C for 60 min without loss of enzymatic activity. The composites were degraded in buffers at 37 °C within 7 days and the average percentage loss in total weight was 5.2%, 92.9%, and 100% for films containing 0.1%, 1.0%, and 5.0% enzyme, respectively. Also, the thicker PCL/AL 1.0% film (10 mm × 10 mm; *h* = ∼500 μm) was degraded within 7 days. Different from the enzyme directly used in previous studies, Tang et al. constructed living plastics by integrating engineered spores inside PCL polymers.^63^ They used specific enzymes (BC-lipase) secreted from genetically programmed *Bacillus subtilis* to break down PCL and engineered spores by inducing spore formation. Living plastics with engineered spores were produced using a 3D printer (120 °C). The incorporation of spores showed no obvious effect on the physical properties of the material. The spore was activated by erosion of the plastic surface, and the subsequent release of BC-lipase from the germinating cells led to almost complete depolymerization of the polymer. The living films depolymerized at an accelerated rate over the operating temperature range of industrial composting facilities and decomposed within 30 days. In contrast, regular films required an additional 20–25 days to break down into pieces. Additionally, the tensile test data showed that there were no discernible differences in the mechanical properties between the regular and living films.

Peñas et al. studied the fabrication and self-degradation of extruded three different polyester films: PLA, PBAT, and PBS, as well as three binary/ternary blends.^64^ To prepare the enzyme-embedded polyesters, polyester powders and *Candida antarctica* lipase B (CalB) powders were blended at different ratios of CalB/polyester (1, 5, and 10 wt %) and then coextruded in a vertical twin-screw MC 5 micromixer at 125 °C for PBS and 170 °C for PLA and PBAT and coblends. The three homopolymers showed different degradation rates, with CalB attacking the PBS film in preference to PBAT and PLA; the autodegraded film of the blends degraded slowly, probably owing to the higher content of PLA and PBAT. For the 1 wt % CalB embedded PBAT and PBS films, similar to all PLA samples, the weight loss curve did not saturate and reach a steady state (linear trend). The degradation rate of the PBS film was found to be almost three times higher: 0.0415%/h (PBS) > 0.0154%/h (PBAT). Similar results were shown when the CalB concentration was increased to 5 wt %, where the degradation rate of the PBS film increased by two folds relative to PBAT (0.6016%/h for PBS and 0.2731%/h for PBAT). Guicherd et al. developed a self-biodegradable enzyme-embedded PLA plastic in which an engineered PLA depolymerase was identified from a thermophilic bacterium, *Actinomadura keratinilytica* T16-1.^11^ The enzyme activity and thermal stability were significantly enhanced by structurally rational engineering, followed by sequential incorporation into PCL and PLA polymers using a melt-extrusion process (70 and 160 °C). The enzyme-embedded PLA films (0.02% w/w enzyme) showed cracks and holes after 8 weeks and were able to completely decompose within the home-composting period of 20–24 weeks. The films also demonstrated great potential in other plastic end-of-life environments, such as industrial compost conditions (58 °C). The control film showed only 68% biodegradation after 90 days; PLA was able to biodegrade at 58 °C, but its biodegradation rate was lower. In the meanwhile, the PLA-depolymerase films reached 91% degradation in 30 days and 100% degradation after 50 days. This was 4.2 times faster than the control group. Anaerobic digestion experiments showed that PLA-depolymerase films achieved 91% carbon conversion within 23 days, whereas biodegradation was not observed in the enzyme-free PLA film after 30 days of incubation. Moreover, the degradation property of the enzyme-embedded film was stable during long-term use and storage. The mechanical test indicated that the addition of PCL resulted in an increase in elongation at break and a slight decrease in tensile strength without any adverse change in Young’s modulus, which was expected because PLA is a type of brittle and stiff polymer that often needs to be modified for application. The perfect match of PLA and PCL improved the overall properties of the materials that would be beneficial for film production and compatible with industrial packaging applications.

In summary, these studies have focused on PCL and PLA, whose degradation can be efficiently catalyzed by several commercially available lipases, such as CalB. PCL and PLA are ideal materials for enzymatic degradation because they are biodegradable, biocompatible, and bioabsorbable, making them particularly suitable for biomedical and environmental applications.^65,66^ The method of delivering the enzyme into BP can be either a solvent-casting or a melt-extrusion process. Extrusion processing typically involves heating a thermoplastic to above or near its melting point, followed by extruding, blowing, or molding the material into a new shape. Therefore, the thermal stability of enzymes is important to maintain activity during the heating process. Strategies such as enzyme engineering, immobilization, spore doping, and 3D printing have been used in the material fabrication. In terms of effectiveness, enzyme-embedded BPs can achieve rapid degradation in the environment, without compromising material properties, which are promising alternatives to the commonly used synthetic polymers. While research and development of enzyme-embedded BPs is still ongoing, there are many critical factors (e.g., enzyme activity, enzyme thermal stability, enzyme formulation, polymer properties, additives) that need to be taken into account when producing these innovative biomaterials.

## Key Factors Affecting the Production of Enzyme-Embedded BPs

### Enzyme Activity

**Figure 4:**
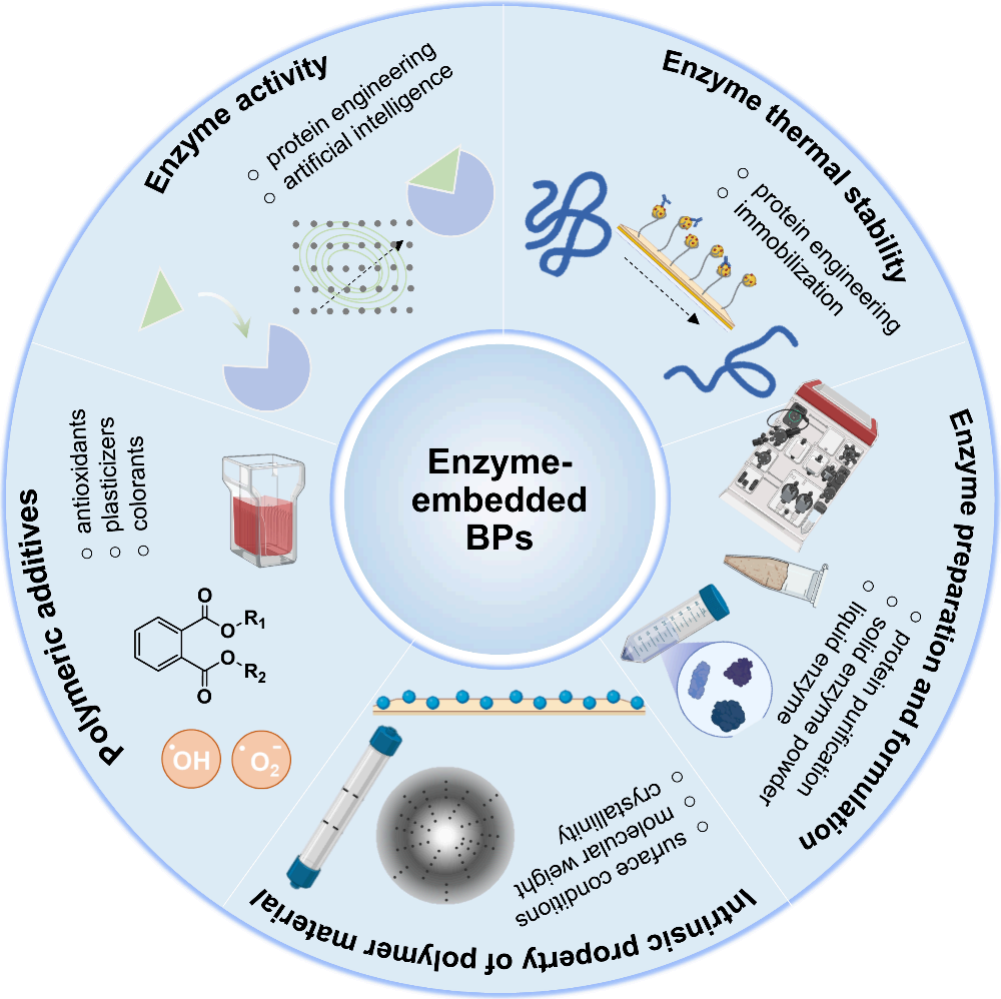
Key factors required to produce enzyme-embedded BPs. They are enzyme activity, enzyme thermal stability, enzyme preparation and formulation, intrinsic properties of polymer material, and polymeric additives.

The degradation activity against BPs must be identified before the enzyme can be used to produce enzyme-embedded BPs (Figure 4). A number of BP-degrading enzymes (e.g., lipase, cutinase, esterase, protease) have been previously discovered from diverse environmental microbes (Table 1). However, the wide-type enzymes often show weak activity, hindering their further application.^67^ To this end, scientists use protein engineering techniques such as rational design and directed evolution approaches to significantly improve the enzyme’s activity.^68^ It is based on either protein sequence or structure to build up a mutant library. Combined with an efficient screening method, some potential enzyme mutants with enhanced hydrolytic activity can be achieved. However, the process of library building and screening sometimes can be quite time-consuming and shows low efficiency for collecting beneficial mutants. Recent advances in artificial intelligence (AI) have made great achievements in the identification of stronger enzyme variants with significantly improved catalytic performance.^69^ For example, machine learning (ML)-driven engineering strategies use the amino acid sequences of enzymes as input data, achieving efficient progression toward the desired function and accelerating optimization of enzyme fitness under several rounds of training and testing.^70,71^ It can accurately quantify the structure–activity relationship for the prediction of diverse enzyme properties. This could potentially offer a fresh, fundamental understanding of the specificity between enzymes and substrates. Furthermore, it is suggested that a combination of experimental library generation and ML-based prediction can improve the efficiency of enzyme engineering efforts.

### Enzyme Thermal Stability

At present, the production of enzyme-embedded BPs mainly involves heat extruding, blowing, and molding (usually more than 100 °C), whereas most natural enzymes have moderate thermal stability. To maintain the enzyme activity during the plastic reconstruction process, these enzymes need to be modified with improved heat tolerance (Figure 4). In general, this can be achieved using protein engineering and immobilization techniques.

(1) Thermophilic proteins are typically considered to have higher overall conformational rigidity than proteins from mesophilic organisms.^72^ Therefore, for protein engineering, there is a need to identify and understand the impact of modifications to flexible regions of the protein structure in order to design thermally stable proteins. The loops are the most flexible regions in proteins, so their rigidification is one of the feasible strategies to improve thermal stability.^73^ Previous studies have showcased methods such as lowering the unfolding entropy to rigidify these flexible loops, deleting or shortening the outer loops, or forming better packing on the protein surface. In addition, the design of surface salt bridges, the introduction of complementary disulfide bonds, and loop anchoring through hydrogen bonding and hydrophobic interactions can also be used to improve the thermal stability of enzymes.^74^

However, experimental methods performed in the laboratory are often time-consuming, expensive, and error-prone, and in some cases, these strategies fail mainly due to insufficient information about protein sequence-structure–function. To achieve this goal, computational design tools can be used to predict the thermal stability of proteins.^75^ Indeed, computational design tools for predictably identifying individual and combinatorial mutations that enhance thermal stability are rapidly evolving and becoming increasingly popular. These developed models are built by training a large number of protein sequences or structures from certain public databases. Combined with machine learning and deep learning algorithms, the accuracy of protein/enzyme thermal stability prediction has been significantly improved. Several reported examples are shown (Table 3).

**Table 3:** Computational Design Tools for Protein Thermostability Prediction

predictor	model mechanisms	availability	ref
TemBERTure	a deep learning package consisting of TemBERTure_DB_, a large-curated database of thermophilic and nonthermophilic sequences, TemBERTure_CLS_, a classifier, and TemBERTure_Tm_, a regression model	https://github.com/ibmm-unibe-ch/TemBERTure	(76)
ThermoLink	collecting disulfide bond and protein thermostability data to construct machine-learning models for the prediction of protein thermostability	guolab.mpu.edu.mo/thermoLink	(77)
mutDDG-SSM	a deep-learning-based framework using the geometric representations encoded in protein structure to predict the mutation-induced protein stability changes	https://github.com/SJGLAB/mutDDG_SSM.git	(78)
convolutional neural network	based on in-depth studies of protein-thermostability-related characteristics and mutation experiment data	not provided	(79)
thermometer	using protein structural information	http://service.tartaglialab.com/new_submission/thermometer_file	(80)
AlphaFold algorithm	combining global conformational sampling with energy prediction using AlphaFold and Rosetta to develop a new computational protocol	https://doi.org/10.5281/zenodo.7497464	(81)
TemStaPro	using embeddings generated by protein language models (pLMs) from an input protein sequence	https://github.com/ievapudz/TemStaPro	(82)
iStable 2.0	integrating 11 sequence- and structure-based prediction tools by machine learning and adding protein sequence information as features	http://ncblab.nchu.edu.tw/iStable2	(83)
innov’SAR	using an innovative sequence–activity relationship methodology based on novel descriptors and digital signal processing to construct a predictive model	not provided	(84)
DeepSTABp	using a transformer-based protein language model for sequence embedding and state-of-the-art feature extraction in combination with other deep learning techniques for end-to-end protein melting temperature prediction	https://csb-deepstabp.bio.rptu.de	(85)
an independently generated data set	an expansion of our previously published data set of mutants for β-glucosidase based on the reported measures of T50 and kinetic constants	not provided	(86)
HotProtein	using a new data set and a novel learning framework for protein thermostability prediction	https://github.com/VITA-Group/HotProtein	(87)

(2) An immobilized enzyme is an enzyme bound to an inert and insoluble substance. There are various methods of immobilizing enzymes: affinity tag binding, adsorption onto alginate beads, substrates or glass, encapsulation, embedding, cross-linking, and covalent bonding.^88^ Immobilized enzymes are better able to withstand extreme conditions such as high temperatures. It also immobilizes the enzyme *in situ* throughout the reaction so that the enzyme can be easily separated from the product and reused in the reaction. This is a very efficient process that is widely used in industries that utilize enzymes to catalyze reactions. The development of enzyme immobilization strategies has contributed to the production of robust and stable custom enzymes. They also enable the recovery and reuse of enzymes, minimize contaminants in the final product, and optimize the control of industrial processes. For example, immobilization is a method of producing ultrastable forms of lipase that can withstand high production temperatures and have a longer shelf life.^89^ Proteases typically have very short half-lives, thus immobilization is required to obtain stable forms of the enzyme and expand and simplify their global applications.^90^ More importantly, it has been documented that embedding immobilized enzymes into the BPs is a viable method to achieve self-degradation. Therefore, the immobilization of enzymes in BPs holds great promise for the development of novel enzyme-embedded BPs.

### Enzyme Preparation and Formulation

From previous studies, it was observed that purified enzymes have been widely used in the production of enzyme-embedded BPs. Certainly, they offer high activity and specificity toward the target substrates, whereas the issues related to the cost of expressing and purifying enzymes remain significant barriers to the implementation of large-scale biomanufacturing. Given the potential benefits, the challenges need to be carefully evaluated to ensure that the transition to enzymatic approaches is feasible and economically viable for the plastics industry. The crude enzymes rather than purified ones could be utilized, but the polymer degradation efficiency might decrease because of host cell proteins in the lysate. Additionally, both solid enzyme powder and liquid enzyme are used to prepare enzyme-embedded materials (Figure 4). However, this seems to be a disputable question. For a majority of previous studies, the solid form of the enzyme was premixed with the polymer particles at different ratios and then joint-extruded to produce the enzyme-embedded polyesters. Inconsistent with that, Guicherd et al. argued that the use of liquid enzyme formulations improved the dispersion of the enzyme in the polymer matrix, as well as the final product’s mechanical properties, compared to solid enzyme formulations.^11^ The reasons to support the introduction of the liquid-form enzyme to prepare an enzyme-embedded polymer are shown below. First, the selection is generally determined by the intended material applications. For example, a fruit or vegetable bag has a thickness of 15 μm, while many other types of packaging bags are multilayered, having only 5-μm-layer thicknesses. These make it impossible to obtain smooth films by introducing the enzyme in powdered or immobilized form. Second, the liquid enzyme can be better distributed inside the polymer. When the enzyme is introduced as a solid powder, there is a discontinuous distribution of the active enzyme as well as the formation of pores on the surface of the material. Third, the liquid environment may provide the optical adaptation for enzymes to carry out the hydrolysis reactions. It simulates the degradation of a substrate by an enzyme in a buffer. Hence, enzyme preparation and formulation are considered to be one of the key factors in the production process of enzyme-embedded BPs.

### Intrinsic Property of Polymer Material

Most conventional petroleum-based plastics such as poly(ethylene terephthalate), polyethylene, polypropylene, polystyrene, and poly(vinyl chloride), are widely used because they are low cost, easily obtained, and have high durability and flexibility. However, they tend to be nonbiodegradable and their increasing accumulation in the environment has posed significant risks to the ecosystems and human health. The petroleum-based BPs such as PCL and PBS can be disintegrated by environmental microbes. While PHAs and PLA are derived from biomass or renewable resources, these are biodegradable and ecofriendly. There are significant differences among these polymers. The intrinsic properties of plastics are responsible for their corresponding biodegradability.^91,92^ More specifically, the surface properties of polymers including specific surface area, hydrophilicity, and hydrophobicity, the primary structure including chemical structure, thickness, molecular weight, and molecular weight distribution, and advanced structure including glass transition temperature, melting temperature, modulus of elasticity, crystallinity, and crystal structure play a critical role in the biodegradation process (Figure 4). For example, enzymes cannot diffuse into the bulk phase of the polymers. As a result, enzyme-mediated decomposition is usually confined to the polymer surface, leading to polymer surface erosion. The surface accessibility related to the hydrophilic or hydrophobic properties is important for enzymes to catalyze the polymer hydrolysis. Moreover, molecules with a maximum molecular weight of less than 1000 Da are readily absorbed by microorganisms. This is much smaller than the typical molecular weight of polymers. Molecular weight is a determining factor in microbial degradation of polymers; the higher the molecular weight, the more difficult it is to biodegrade. Similarly, microbial degradation of lower molecular weight takes less time. It is essential to break down polymers into small molecular-weight oligomers for microbial utilization. In addition, it has been reported that enzyme-mediated ester hydrolysis increases with decreasing glass transition temperature, *T*_g_, for both amorphous and semicrystalline polyesters. For semicrystalline polyesters, enzymatic hydrolysis was negatively correlated with the melting temperature, *T*_m_. Both dependencies reflect the restricted mobility of polyester chains in the glass domains and grain layers, which hinders the formation of enzyme–substrate complexes and thus the hydrolytic breaking of ester bonds.^93^

### Polymeric Additives

During the production of polymer plastics, various additives such as antioxidants, plasticizers, and colorants (pigments or dyes) as well as other compounds are incorporated to enhance the polymers’ performance in terms of thermal and mechanical properties, and even color characteristics and overall appearance^94^ (Figure 4). Although the use of additives in conjunction with polymers is optimized, they may introduce additional challenges during polymer recycling. Several toxic plasticizer additives such as phthalate esters (PAEs) can be released from the plastic degradation process, causing secondary pollution. This limitation has an impact on the potential applications of recycled polymers and leads to environmental pollution. Fortunately, the incorporation of some additives, such as pro-oxidants, natural polymer compounds, nonionic surfactants, CaCO_3_, mineral oils, etc., can contribute to the exposure of plastics to microorganisms, thus triggering and enhancing the biodegradation of plastics.^95,96^ An enzyme cocktail for the effective biodegradation of mixed plastics and additives could be an interesting direction for future study. Since no single enzyme is capable of degrading multiple types of plastics and additives, formulating mixtures of relevant enzymes can help to effectively biodegrade multiple plastic wastes at the same time.

### Challenges in the Development and Application of Enzyme-Embedded BPs

The enzyme-embedded biodegradable materials are an innovative update of classical BPs. An increasing number of studies have reported the development and characterization process and showed the huge potential of enzyme-embedded BPs in environmental remediation applications. Through embedding enzymes into biodegradable polyesters such as PLA and PCL, the higher degradation efficiency has been confirmed under both industrial and home composting conditions. These enzyme-embedded plastics have been claimed to be highly efficient, sustainable, and cost-effective, without generating secondary environmental contamination and/or biosafety concerns. However, there are still several challenges to be overcome before they can be widely used.

(1) Currently, a hot-melt-extrusion process is usually used for the installation of an enzyme into the polymer when developing enzyme-embedded BPs. This is particularly effective for low-melting-point polymers [e.g., PLA (160 °C) and PCL (60 °C)]. Nevertheless, introducing a specific enzyme into higher-melting-point polymers [e.g., polyamide (230–290 °C), poly(ethylene terephthalate) (260–280 °C), poly(butylene terephthalate) (240–275 °C), and polycarbonate (280–320 °C)] remains a challenge. The challenge partly comes from the utilization of heat during plastic processing. Most enzymes are denatured at high temperatures and completely lose their biological activity. One possible solution to this issue is the use of additional protective matrices around the enzyme, i.e., immobilization. Several previous studies have demonstrated the effectiveness of this method.

(2) For the solvent-cast process to produce enzyme-embedded BPs, three key aspects need to be considered. The first is the effect of solvent on the enzyme activity and stability. Many organic solvents have been reported to negatively affect the enzyme’s catalytic performance. This should be evaluated before the production of solvent-cast composites. The second is the effect of solvents on the surrounding environment and human health. Since toxic organic solvents such as chloroform are often used, exposure to these hazardous chemicals would lead to serious environmental and health problems. The third is the possibility of the solvent-casting method to be applied in the industrial production of enzyme-embedded BPs. Industrial costs, processes, environment, and management of personnel need to be considered.

(3) A full consideration of both enzyme and polymer properties is required during the production of enzyme-embedded BPs. For example, PBS has a low melting point at 115 °C, while few enzyme-embedded PBS have been reported. This may be due to its crystalline property; it degrades slowly in nature. Moreover, to achieve a new material with various physical properties, polymer blends and composites are often produced in industry. How to install active enzymes into these blending polymers to improve their biodegradability and compostability is a challenging, but interesting question.

(4) The biodegradation efficiency of enzyme-embedded BPs is often not as high as expected. The major limitation is associated with the insufficient activity of the embedded enzyme. Moreover, introducing the powder-form enzyme into the BPs may result in poor distribution, loss of enzyme activity during the heat process, and more importantly, prevent its production of thin-wall packages. In contrast, the liquid enzyme is compatible with the microenvironment where the enzyme can perform its optimal activity and stability. Yet, this could lead to more plastic processing steps involved to ensure the enzymatic degradation activity against polymer from the inside part.

(5) Although several enzyme-embedded BPs have been successfully developed in previous studies, the underlying reaction mechanism of embedded enzymes remains poorly understood. After assembling the active enzyme molecules into the polymers, how to degrade these macromolecules from inside to outside, how to facilitate substrate accessibility in both solid-state and liquid-state enzymology, and how to release products from the active center are elusive questions. Addressing these issues could advance the development of enzyme-embedded composites for sustainable application.

(6) The research and development of enzyme-embedded BPs are still at the laboratory level. It remains far behind the industrial and environmental applications. When scaling production, more practical factors are required to be considered for the enzyme, polymer, and the produced composite, such as processing conditions, compatibility with the industrial environment, transport, and storage. The precise management of raw materials, cost, and time is also required. The life cycle assessment measures are needed to assess the whole process of BP production, recycling, and its effect on the environment.

### Conclusions and Prospects

BPs have gained increasing attention in recent years owing to their potential biodegradability and recyclability. They are considered one of the most effective methods to slow down plastic pollution. However, biodegradation of BPs depends on certain environmental conditions and the micro- and nanoplastics generated from BPs are emerging environmental contaminants. BPs may be a part of the solution, but there is still a long way to go to solve the global plastic pollution dilemma. At this point, enzyme-embedded BPs emerging as innovative biomaterials can achieve rapid biodegradation and compostability at ambient temperature, without compromising material properties. They meet both the home-compost and industrial process standards, representing a novel and practicable approach to tackling plastic degradation in a virtuous carbon circle. While the development of enzyme-embedded BPs is in its infancy, more efforts are needed to achieve large-scale production and sustainable application. For this goal, more quantities and different types of enzymes as well as different polymer matrices can be experimented with in the preparation of enzyme-embedded plastics. These versatile combinations could provide more opportunities for the development of advanced functional biomaterials. It will save a lot of time and cost if efficient, stable, green, and intelligent plastic processing pipelines can be formed. In particular, the computational tools can be used to optimize the processing parameters (e.g., temperature, pH, ratios of enzyme and polymer) in industry. Altogether, at this critical intersection of biotechnology and sustainable materials, the enzyme-embedded BPs represent a new type of promising innovative biomaterial that not only addresses immediate environmental concerns but also contributes to more sustainable commercial plastic life cycles.
